# Perception of otherness - the role of personality and demographic variables

**DOI:** 10.1192/j.eurpsy.2021.999

**Published:** 2021-08-13

**Authors:** M. Preiss, N. Doubková, J. Jonáš

**Affiliations:** 1 Psychology, University of New York in Prague, Praha, Czech Republic; 2 Psychology, National Institute of Mental Health, Klecany, Czech Republic

**Keywords:** otherness, personality functioning, social distance

## Abstract

**Introduction:**

The idea that personality can influence our perception of ‘otherness’ is widely accepted within the literature of social sciences. Undoubtedly, the principle of dehumanization played an important role in genocides during the 20^th^ and 21^st^ centuries. In totalitarian or post-totalitarian regimes ‘otherness’ may present a challenge to the absolute power. Recent studies showed that negative attitudes toward ‘otherness’ – also known as xenophobia – are on a rise in the Czech Republic.A deeper analysis of the personality in relation with perception of otherness is still missing.

**Objectives:**

The presentation analyse the personality variables associated with the perception of otherness and compare the differences between various age groups, genders, individuals with different levels of education, and above all, the differences between various groups. Several contrast groups are compared - general population, high neuroticism sample, personality disorder sample, xenophobic and xenophilic sample.

**Methods:**

Bogardus Scale of Social Distance as a measure of perception of otherness is compared with in-depth analysis of personality functioning (Semi-Structured Interview for Personality Functioning DSM–5, STiP-5.1).

**Results:**

We analyze the results of five samples with respect to demographic variables, variables of personality functioning and try to point out the relationship between more attitudes and underlying personality functioning. The importance of some demographic variables (as age) and connections between personality functioning (Self and Interpersonal) and social distance is emphasized and discussed.

**Conclusions:**

The project help us to understand perception of otherness in light of demographic and relative power of personality factors.

